# Mechanical and Anti-Icing Properties of Polyurethane/Carbon Fiber-Reinforced Polymer Composites with Carbonized Coffee Grounds

**DOI:** 10.3390/ma18194533

**Published:** 2025-09-29

**Authors:** Seong Baek Yang, Min Ji Woo, Donghyeon Lee, Jong-Hyun Kim, Sang Yong Nam, Dong-Jun Kwon

**Affiliations:** 1 Research Institute for Green Energy Convergence Technology, Gyeongsang National University, Jinju 52828, Republic of Korea; sbyang@gnu.ac.kr (S.B.Y.);; 2 Department of Polymer Science and Engineering, School of Materials and Engineering, Gyeongsang National University, Jinju 52828, Republic of Korea; 3 Department of Materials Engineering and Convergence Technology, Gyeongsang National University, Jinju 52828, Republic of Korea

**Keywords:** carbonized spent coffee ground, polyurethane, sustainable filler, carbon fiber-reinforced polymer

## Abstract

Spent coffee grounds represent an abundant waste resource with potential for sustainable material applications. This study investigates the use of carbonized spent coffee grounds (CSCG) as fillers in polyurethane (PU) coatings for carbon fiber-reinforced polymer (CFRP) substrates to enhance mechanical durability and anti-icing performance. SCGs were dried, sieved (<100 µm), and oxidatively carbonized in air at 100–300 °C for 60–120 min, then incorporated into PU at 1 or 5 wt.% and applied by spray-coating. A full-factorial design was employed to evaluate the effects of carbonization temperature, particle size, and filler loading. The optimized formulation (300 °C, 100 µm, 5 wt.%) showed the highest water contact angle (103.5°), lowest work of adhesion (55.8 mJ/m^2^), and improved thermal stability with 60% char yield. Mechanical testing revealed increased tensile modulus with reduced strain, and differential scanning calorimetry indicated an upward shift in glass-transition temperature, suggesting restricted chain mobility. Ice formation at 0 °C was sparse and discontinuous, attributed to lowered polar surface energy, rough surface texture, and porous carbon morphology. These results demonstrate that CSCGs are effective sustainable fillers for PU coatings, offering combined improvements in mechanical, thermal, and anti-icing properties suitable for aerospace, wind power, and other icing-prone applications.

## 1. Introduction

In contemporary society, coffee has become one of the most widely consumed beverages globally. It has been reported that more than 1 billion people worldwide drink coffee daily, with 73% of consumers drinking coffee every day [1]. Approximately 2.25 billion cups of coffee are consumed each day, and around 10.3 billion kilograms of coffee were consumed globally in 2022–2023 [2,3]. Proportional to this high consumption, substantial amounts of spent coffee grounds (SCGs) are generated. It is estimated that, annually, between 6 million and 60 million tons of coffee grounds are produced worldwide. Notably, of the 15 g of coffee beans typically used to prepare a single cup of Americano, approximately 14 g (99.8%) end up as waste [3].

Traditionally, the majority of SCGs generated have been disposed of in landfills or incinerated without specific reuse strategies. Such practices pose significant environmental concerns, as the anaerobic decomposition of coffee grounds in landfills releases potent greenhouse gases, notably methane (CH_4_) and carbon dioxide (CO_2_) [4,5,6]. CH_4_ exhibits a greenhouse effect approximately 28–34 times stronger than CO_2_, and even when incinerated, approximately 338 kg of carbon per ton of coffee waste is emitted, thereby contributing to climate change [7]. On the other hand, the recycling and valorization of SCG offer significant environmental benefits. These approaches can markedly reduce methane emissions and alleviate the demand for landfill space, consequently conserving valuable land resources. However, recycling processes inevitably involve conflicting aspects, including the energy consumption required for reprocessing and the necessity of additional materials. Life cycle assessment studies have revealed that the thermal treatment of SCG is environmentally more sustainable compared to traditional petroleum-based synthesis methods [8]. Nonetheless, continuous research and broader societal consensus are needed to fully realize and optimize these benefits.

SCGs are a representative cellulose-based lignocellulosic biomass. They are primarily composed of cellulose (8.6–23.8%), hemicellulose (16.7–39.1%), lignin (23.9–28.6%), proteins (17.4–18.7%), and lipids (2.3–3.8%) [9,10,11]. Due to their structural similarity to other plant-based materials, SCGs undergo a transformation into porous materials upon oxidative thermal treatment in air, which effectively removes organic residues and volatile substances originally present within their structure. The porous structure formed through this oxidative process is characterized by a high specific surface area and provides a practical pathway for waste upcycling. Such cellulose-based porous materials can offer significant advantages in polymer/filler composite applications, providing structural characteristics essential for functional performance. Recently, increasing attention has been directed toward the use of sustainable composites and bio-based fillers for functional coatings, where biomass-derived wastes are explored as alternative resources. For instance, recent studies have demonstrated the potential of waste valorization strategies for enhancing polymer composite properties and surface functionalities [12,13]. These developments align with the objectives of the present work, which seeks to upcycle spent coffee grounds (SCGs) into functional fillers for polyurethane (PU) coatings with anti-icing capabilities.

Among the various thermal treatment approaches, this study employed a straightforward oxidative thermal treatment method that controls temperature alone. Unlike conventional carbonization, which is designed to prevent combustion and promote char formation, our method induces partial oxidation and decomposition, leading to the formation of porous bio-carbon with surface functionalities. This distinction is critical for interpreting the observed transformations and positioning CSCG as an upcycled filler material. This distinction is critical for interpreting the resulting chemical and structural transformations. Thermal pyrolysis or carbonization is particularly attractive as an environmentally friendly process since it generally does not require solvents or hazardous reagents. Unlike conventional recycling methods involving extraction or chemical modifications—which typically require substantial amounts of organic solvents and reagents—thermal pyrolysis converts SCG into carbonaceous materials using heat alone, thereby preventing the generation of additional chemical waste. However, one notable disadvantage of thermal methods is the inherent energy consumption due to the use of heat. For example, activation energies measured during the pyrolysis of coffee shells at heating rates of 5, 10, and 20 K/min were reported as 17.2, 18.0, and 57.5 kJ/mol, respectively [14]. However, there is potential to significantly enhance the overall energy efficiency of the process if the combustible gases generated during pyrolysis are recovered and reutilized as an energy source.

The research team explored specific application areas where oxidatively carbonized spent coffee grounds (CSCGs) could be effectively utilized as composite material fillers. As a result, it was decided to focus on aerospace and extreme weather conditions related to climate change, particularly addressing icing issues, which have recently gained considerable attention. Ice accumulation on surfaces under harsh environmental conditions poses severe threats to the safety of aircraft and critical infrastructure. Specifically, carbon fiber-reinforced polymer (CFRP) composites exhibit relatively low thermal conductivity and are vulnerable to overheating; thus, anti-icing systems must be designed carefully. Utilizing CSCGs as cellulose-based fillers for anti-icing applications offers several advantages. Due to their high specific surface area, rough surface morphology, and reduced polar surface energy, these fillers can suppress continuous ice nucleation, leading to sparse and discontinuous ice coverage (anti-icing effect), thereby reducing ice adhesion and facilitating easier removal during thawing periods. Indeed, a coating composed of biochar/polypyrrole demonstrated an ice adhesion strength of 65.0 kPa, approximately a 65% reduction compared to an uncoated surface (184.7 kPa). Moreover, the photothermal conversion properties inherent in oxidatively carbonized coffee grounds can provide active anti-icing functionality through efficient solar absorption and localized heating.

Bio-waste materials, particularly CSCG, significantly contribute to anti-icing performance through their capability to inhibit ice nucleation. Biological materials exhibit varied influences on ice nucleation; while some can enhance ice formation, suitably processed biomaterials may suppress nucleation. Surface roughness at critical dimensions plays an essential role in inhibiting ice nucleation. For example, the critical stable ice nucleus size at −25 °C has been identified as approximately 1.7 nm [15]. The porous structure and surface roughness of CSCG are thus expected to effectively suppress ice nucleation by addressing this critical size criterion. Furthermore, bio-inspired antifreeze protein-mimetic coatings have demonstrated a remarkable capacity to depress ice nucleation temperatures down to −29.4 °C and limit ice propagation rates to below 0.00048 cm^2^/s [16]. This suggests that properly engineered biomaterials such as oxidatively carbonized SCG have considerable potential for enhancing anti-icing functionalities in practical applications. Also, surfaces featuring hierarchical or microstructured textures with high specific surface areas create air pockets between water droplets and the surface, significantly reducing the actual ice-to-surface contact area. Consequently, ice adhesion is markedly diminished, facilitating easier ice removal. For instance, superhydrophobic surfaces with water contact angles exceeding 150° can exhibit ice adhesion strengths more than nine times lower compared to conventional surfaces [17,18]. Moreover, surfaces characterized by high surface areas and rough structures help water droplets retain their spherical shape, delaying freezing and promoting effortless detachment of ice during thawing periods [19].

In this study, the design of experiments (DoE) methodology was employed to systematically investigate the optimal formulation and process conditions for polyurethane (PU) coatings filled with SCG. DoE is a widely adopted experimental approach for identifying the relationships between factors affecting experimental outcomes by systematically varying the parameters and evaluating the resulting responses, thereby significantly reducing the total number of required experiments [20]. The use of DoE not only decreases experimental effort, leading to cost savings in material consumption and reduction in waste, but also enables the prediction of outcomes based on statistical analysis. Additionally, DoE offers deeper insights into the interactions between various factors, facilitating future scale-up processes and enabling automation. In composite material development, DoE allows the determination of optimal conditions with minimal experiments and supports the establishment of statistically validated predictive models. Thus, this approach was applied in the current research to establish optimal conditions for applying PU and SCG-based filler coatings on composite surfaces.

Ultimately, this research utilized oxidative thermal treatment to convert discarded SCGs into cellulose-based fillers, which were subsequently mixed with PU to produce a dispersion solution. This solution was spray-coated onto CFRP substrates. The resulting coatings were systematically evaluated for their mechanical properties, thermal characteristics, and anti-icing performance, employing a spraying technique for consistent and repeatable application. Rather than claiming full mechanical durability at this stage, we present these observations as early indicators of performance potential, highlighting the feasibility of CSCGs as sustainable fillers. Currently, petroleum-based polyurethane and environmentally harmful solvents are utilized; however, the study indicates promising prospects with the potential application of automated spraying systems, substitution with bio-based polymers, and replacement by bio-solvents [21,22]. Specifically, developing bio-polyols derived directly from coffee waste and waterborne PU systems could pave the way towards fully bio-based, low-emission composite coatings. This integrated approach simultaneously achieves three critical objectives: waste valorization, environmental protection, and the development of high-performance functional coatings. Particularly aligned with circular economic principles, this innovative solution is expected to significantly advance sustainable anti-icing applications in aerospace and wind-energy industries.

## 2. Materials and Methods

### 2.1. Materials

SCGs were collected from Starbucks Korea after brewing Espresso Roast beans and used as received without any further pretreatment. A tow-component acrylic PU coating system (Spiroltan; Samhwa Paint industrial Co., Ltd., Seoul, Republic of Korea) was employed as the polymer matrix, consisting of the base resin (Part A: poly [2-methyl-2-propenoic acid-co-butyl 2-propenoate-co-styrene-co-2-hydroxyethyl 2-methylacrylate] in *n*-butyl acetate/toluene, 40 wt.%; MSDS No. 13230733-2) and an isocyanate hardener (Part B; hexamethylene diisocyanate homopolymer, 60 wt.% in ethylbenzene/xylene; MSDS No. 13230732-3). The coating system was diluted as needed with Uniall thinner, spring-fall grade; MSDS No. 13120181-100). All coating components were used without further purification. CFRP substrates were fabricated from a 2 mm-thick twill-weave carbon- fiber fabric prepreg based on T-700 fibers (Toray, Nagoya, Japan; supplied by Hankook Carbon, Pohang, Republic of Korea). The laminates were cut into 16 mm × 80 mm coupons for subsequent anti-icing tests.

### 2.2. Preparation of CSCG

**Figure 1:**
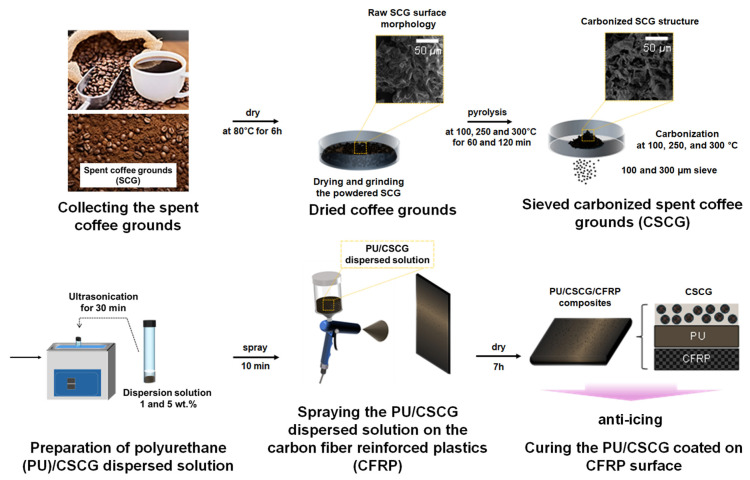
Process flow of PU/CSCG composite fabrication and property evaluation through carbonization, grinding, mixing, and coating of SCG.

SCGs were first dried at 80 °C for 6 h in a forced-convection oven (OF-22, Jeiotech, Daejeon, Republic of Korea) to remove residual moisture. The dried residue was then sieved to a particle size below 100 µm. Subsequently, the pre-dried SCGs were subjected to oxidative thermal treatment in air using a muffle furnace (MF-12G, Jeiotech, Daejeon, Republic of Korea) at three set temperatures—100 °C, 250 °C, and 300 °C—for holding periods of 60 min and 120 min, respectively. After oxidative thermal treatment, the powders were classified through 100 µm and 300 µm sieves to obtain uniform fractions (Figure 1). The resulting samples were designated as CSCG-T-t, where T is the oxidative thermal treatment temperature (°C) and t is the dwell time (min).

### 2.3. Fabrication of PU/CSCG Composite Coating

CSCGs were dispersed into a PU solution—composed of 5 mL Spiroltan A, 1 mL Spiroltan B, and 0.67 mL Uniall Thinner—at loadings of 1 or 5 wt.%. The mixture was ultrasonicated (275DAE, Crest, New Jersey, America) for 30 min to break up agglomerates and obtain homogeneous dispersion. The resulting PU/CSCG suspension was spray-coated onto CFRP coupons (16 × 80 mm) from a stand-off distance of 150 mm for approximately 10 min and then dried at 23 ± 2 °C and 50 ± 5% R.H. for 7 h to form the first coating layer. For each specimen, a PU primer was separately prepared by blending 5 mL Spiroltan A, 1 mL Spiroltan B, and 0.67 mL Uniall Thinner and was sprayed onto the CFRP substrate at the same 150 mm distance. This primer remained tack-free for roughly 10 min. During this interval, CSCG powder (1 or 5 wt. % relative to the total liquid) was dispersed in 3 mL of fresh Uniall Thinner using an ultrasonic bath (275DAE, Crest, Ewing Township, NJ, USA) for 3 min. At the 10 min mark, the well-dispersed PU/CSCG suspension was sprayed over the still-tacky primer under identical conditions, ensuring intimate interlayer bonding. Finally, all coated specimens were cured for 24 h at 23 ± 2 °C and 50 ± 5% R.H. prior to mechanical, thermal, surface-property, and anti-icing evaluations (Figure 1).

### 2.4. Design of Experiments

A full-factorial design was adopted with 3 independent factors: carbonization temperature (A: 100, 250, 300 °C), particle size (B: 100 µm, 300 µm), and filler loading (C: 1 wt.%, 5 wt.%), giving 3 × 2 × 2 = 12 experimental runs. The response variables were tensile strength, tensile modulus, and glass-transition temperature (T_g_). Main and interaction effects were evaluated through main-effect plots and Pareto charts, and statistical analyses, including regression modeling, were performed using Design-Expert version 13 (Stat-Ease, Minneapolis, MN, USA) [23,24].

### 2.5. Interface-Energy-Driven Deicing Mechanism

Coating thickness was measured with a high-precision gauge (CM8811FN, Nicety, Shenzhen, China). Static contact angles (θ) were determined using a digital microscope (Dino-Lite AF4915ZT, Dunwell Tech, Torrance, CA, USA) with 10 µL droplets of de-ionized water, formamide, ethylene glycol, and diiodomethane; five replicate measurements at the same spot were averaged for each liquid.

The dispersive ($\gamma_{S}^{d}$) and polar ($\gamma_{S}^{p}$) components of the solid surface energy were calculated from the Owens–Wendt–Rabel–Kaelble (OWRK) model combined with Young’s equation:

(1) $$\gamma_{L}\left(1+\cos\theta \right)=2(\sqrt{\gamma_{S}^{d}\gamma_{L}^{d}}+\sqrt{\gamma_{S}^{p}\gamma_{L}^{p}})$$

(2) $$\gamma_{S}=\gamma_{S}^{d}+\gamma_{S}^{p}$$

where $\gamma_{L}$, $\gamma_{L}^{d}$, and $\gamma_{L}^{p}$ denote the total, dispersive, and polar tensions of each probe liquid, respectively [25].

Using the resolved surface-energy components, the work of adhesion between water and the coating was determined from the Young-Dupré relation,

(3) $$W_{ad}=\gamma_{L}(1+\cos\theta )$$

so that lowering $\gamma_{S}^{p}$ (and hence increasing θ) directly reduces and weakens ice binding [26].

Porosity introduced by the CSCG filler decreases the real contact area, promoting the Cassie–Baxter wetting regime described by

(4) $$\cos\theta_{C}=f_{s}\cos\theta_{Y}+(1-f_{s})$$

where $\theta_{C}$ is the apparent contact angle, $\theta_{Y}$ the intrinsic angle, and $f_{s}$ the fractional solid–liquid contact area [27]. Lower $f_{s}$ values enlarge $\theta_{C}$, enhance hydrophobicity, and maintain an insulating air layer that disrupts both ice nucleation and growth. Thus the PU/CSCG coatings achieve outstanding anti-icing performance through the combined effects of (i) a near-zero polar surface-energy component that minimizes $W_{ad}$ and (ii) hierarchical roughness that lowers $f_{s}$ and sustains a Cassie–Baxter state.

### 2.6. Characteristics

Comprehensive characterization encompassed coating-thickness, microstructural, thermal, chemical, surface, mechanical, and anti-icing evaluations. Film thickness was measured with a precision gauge (CM8811FN, Nicety, Shenzhen, China). The surface and internal morphologies of SCG and CSCG were observed by SEM (SEM, JSM-7600, JEOL, Tokyo, Japan) to assess pore formation, roughness, and particle fragmentation after the various thermal treatments. Thermal stability and decomposition behavior were compared by thermogravimetric analysis and derivative thermogravimetric analysis (TGA and DTG) (N_2_, room temperature → 600 °C, 10 °C min^−1^), and T_g_ was obtained from differential scanning calorimetry (DSC). Chemical evolution was tracked by Fourier transform infrared (FT-IR) (4000–600 cm^−1^), and crystallinity/turbostratic ordering by X-ray diffraction (XRD, Cu Kα). Static contact angles with de-ionized water, ethylene glycol, and di-iodomethane were converted to dispersive and polar surface-energy components via the Owens–Wendt–Rabel–Kaelble model, and the Young–Dupré equation furnished the work of adhesion. Tensile strength and modulus were determined on a universal testing machine (AG-100kNX plus, Shimadzu Co., Ltd., Kyoto, Japan) in accordance with ASTM D638 [28]. Anti-icing performance was quantified in a climate box held at 13 °C and 80% R.H.: coated CFRP coupons (16 × 80 mm) were placed on the lid of an aluminum chamber thermally coupled to a refrigerated circulator bath set to −80 °C (RW-3040G, Jeiotech, Cheongju, Republic of Korea) to maintain a constant sub-zero surface. Top- and side-view images of ice formation were captured for 30 min with a digital microscope (Dino-Lite AF4915ZT, Dunwell Tech, Torrance, CA, USA [29]). Ice nucleation was evaluated by determining the iced area within a 10 mm × 10 mm window at three positions per sample and averaging the values to minimize error, providing quantitative ice-coverage, density, and thickness data for correlation with the interface-energy metrics.

## 3. Results and Discussion

### 3.1. Morphological Evolution of SCG Under Thermal Treatment

**Figure 2:**
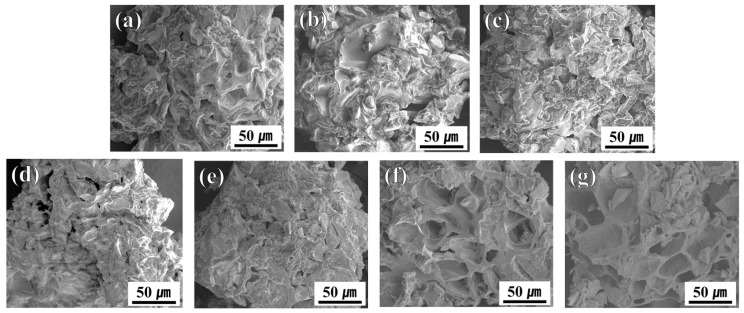
SEM images of spent coffee grounds and carbonized spent coffee grounds prepared under different thermal treatment conditions: (**a**) SCG (untreated); (**b**) CSCG-100-60; (**c**) CSCG-100-120; (**d**) CSCG-250-60; (**e**) CSCG-250-120; (**f**) CSCG-300-60; (**g**) CSCG-300-120.

Figure 2 shows SEM images of SCG and CSCG prepared under different thermal treatment conditions. Figure 2a represents untreated SCG, exhibiting a generally smooth surface texture with sporadic, irregularly shaped pores measuring approximately 20–30 µm. The SCG particles distinctly show the characteristic wrinkled and folded morphology typical of coffee grounds, with individual particle sizes generally exceeding 200 µm. The study conducted by Mohammed et al. also inferred that the SCGs exhibit a certain degree of porosity even before undergoing alkali treatment, which contributes to an increase in specific surface area [30]. The research team led by Majed also noted that the SCG used in their study exhibited a denser, more irregular, and rougher morphology [31]. In the CSCG samples subjected to thermal treatment (Figure 2b–g), noticeable morphological changes are evident according to variations in treatment temperature and duration. Under thermal conditions of 100 °C (Figure 2b,c), the overall original morphology is relatively preserved, displaying only minor alterations and maintaining a comparatively smoother texture. In contrast, at 250 °C (Figure 2d,e), a substantial increase in surface roughness is observed, and the pores become more distinct and pronounced. Moreover, unidentified residual materials filling the pores are prominently visible at this temperature, suggesting that pore formation is linked to the vaporization and release of volatile materials present within the coffee grounds at higher temperatures. This interpretation is based on a series of reactions that occur during the conversion of oxidatively carbonized biomass, including the release of volatile compounds, the decomposition and reorganization of hemicellulose and cellulose polymer rings, the carbonization of methoxy and aliphatic groups, and the repolymerization and dehydration of lignin [32,33,34]. At 300 °C (Figure 2f,g), the CSCG displays clearly developed porous structures with expanded open-type pores reaching approximately 50 µm in diameter. This condition shows the most distinctively dry and rough surface texture, implying that volatile organic substances and other internal compounds have been thoroughly volatilized, resulting in pronounced open-pore structures [35]. However, increasing thermal treatment duration from 60 to 120 min at constant temperature did not result in significant morphological changes. Thus, it can be concluded that the morphological characteristics of oxidatively carbonized spent coffee grounds are more sensitive to treatment temperature rather than duration, especially highlighting significant structural transformations above 250 °C. These morphological evolutions not only enhance porosity and surface roughness but also play a crucial role in reducing surface energy and suppressing continuous ice nucleation, thereby contributing directly to the anti-icing functionality of CSCG. Moreover, such structural transformations demonstrate the feasibility of upcycling waste coffee grounds into functional fillers for sustainable surface protection applications.

### 3.2. Thermal Decomposition Behavior (TGA/DTG) of SCG and CSCG

**Figure 3:**
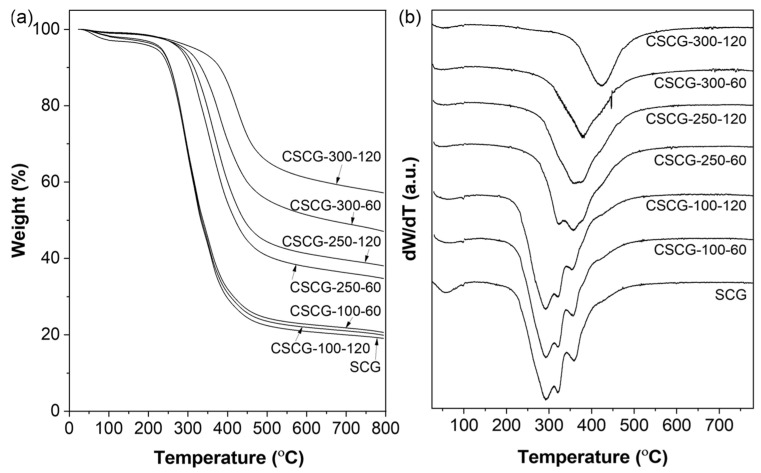
Thermal behavior of SCG and CSCG under different thermal treatment conditions: (**a**) TGA curves showing overall weight loss trends; (**b**) DTG curves indicating decomposition peaks.

The TGA and DTG analyses presented in Figure 3 clearly depict the thermal decomposition behavior of untreated SCG and CSCG under various oxidative thermal treatments (100 °C, 250 °C, and 300 °C for 60 and 120 min). Numerous studies on TGA and DTG analyses of SCG have been reported. In one study, SCG was heated up to 905 °C, and the mass loss and composition of volatile products were analyzed, confirming that approximately 75 wt.% of volatile substances were released [36]. Additionally, Fermoso and Mašek (2018) investigated the thermochemical decomposition process of SCG using TG-MS analysis, identifying the order of reactivity for biopolymer components as hemicellulose > cellulose > lignin [37]. For untreated SCG, examining the results of this study, the prominent DTG peak around 250–300 °C corresponds to the significant thermal degradation of hemicellulose and partially cellulose [38,39]. Morphologically, small folded pores (~20–30 µm), observed in Figure 2a, likely formed due to the release of volatile organic compounds, such as acetic acid and furfural, during initial decomposition. At mild thermal treatment condition (100 °C), CSCG retained most original biomass constituents, reflected by minimal morphological changes and almost unchanged TGA curves (Figure 2b,c). The preservation of folded pores and smooth textures supports the conclusion that organic constituents were not significantly decomposed at this temperature. CSCG treated at 250 °C showed partial degradation of hemicellulose and some remaining cellulose, causing further decomposition in subsequent TGA analysis. Morphological analysis (Figure 2d,e) supports these results, indicating expanded pores partially filled with unidentified residuals, confirming partial pyrolysis and volatilization at this intermediate temperature. For CSCG subjected to oxidative thermal treatment 300 °C, approximately 60 wt.% residual weight observed in the TGA curve represents charred carbon and ash formed after extensive decomposition of biomass organic constituents [40]. The main decomposition temperature range of cellulose can generally be regarded as between 300 and 350 °C, although some studies have reported a DTG peak at approximately 307 °C. Research on cellulose insulation materials has also indicated decomposition starting between 250 and 260 °C, reaching a maximum at around 301.5 °C. The finding that coffee grounds carbonized at 300 °C exhibit a residual mass of about 60 wt.% implies that carbon and inorganic ash are the primary constituents remaining after the carbonization process of coffee by-products [41,42]. The nearly vanished DTG peaks confirm the extensive removal of unstable volatile matter at this higher temperature. Correspondingly, SEM images (Figure 2f,g) reveal clearly expanded open-type pores (~50 µm), resulting from the complete gasification of internal organic compounds. Such morphological features offer enhanced thermal stability and structural advantages when incorporated as fillers in composites. These thermal and morphological evolutions reduce polar surface functionalities and lower surface energy, suppressing continuous ice nucleation and thereby contributing directly to the anti-icing functionality of CSCG. Collectively, these integrated results demonstrate both the correlation between thermal decomposition and morphological changes and the feasibility of upcycling waste coffee grounds into sustainable fillers for advanced anti-icing composite applications.

### 3.3. Chemical and Structural Evolution of SCG During Carbonization

**Figure 4:**
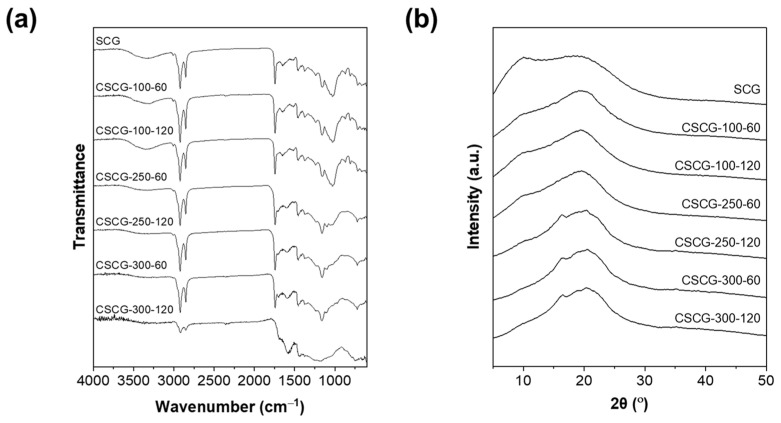
Spectroscopic and structural characterization of SCG and CSCG under different thermal treatment temperatures: (**a**) FTIR spectra showing changes in surface functional groups; (**b**) XRD patterns indicating structural transitions and incipient turbostratic ordering.

As shown in Figure 4a, the chemical transformations of SCG during oxidative thermal treatment from 100 °C to 300 °C are reflected in the FTIR spectra, which reveal the sequential reduction and rearrangement of surface functional groups. At 100 °C, the broad O–H stretching band spanning the range of 3200–3600 cm^−1^ dominates the spectrum, indicating the combined presence of cellulose, hemicellulose, and adsorbed moisture. The broad O–H stretching vibrations observed in the range of 3200–3600 cm^−1^ were identified as originating from intermolecular and intramolecular hydrogen bonds within cellulose and hemicellulose, as well as from adsorbed moisture. The hydrogen bonding network is a critical factor determining the crystalline structure of cellulose, and the O–H stretching vibrations exhibit high coupling and delocalized characteristics through intrachain and interchain hydrogen bonds. The strong O–H band observed at 100 °C serves as an indicator of the structural integrity of polysaccharides [43,44,45]. At this temperature, strong and distinct aliphatic –CH_2_– and –CH_3_ stretching vibrations between 2800 and 3000 cm^−1^ confirm that aliphatic groups within the carbohydrate structure largely remain intact. Asymmetric and symmetric stretching vibrations of the –CH_2_– groups are observed at 2924 cm^−1^ and 2854 cm^−1^, respectively, indicating the presence of fatty acid structures and carbohydrate backbones within the coffee grounds. The reduction in these bands with increasing temperature suggests the progression of dehydration and dehydrogenation reactions [46,47]. A pronounced carbonyl (C=O) stretching peak around 1730 cm^−1^ further suggests the retention of hemicellulose and lignin-derived carbonyl groups. Moreover, intense C–O–C vibrations observed in the 1000–1200 cm^−1^ region indicate that the polysaccharide backbone has not yet experienced significant cleavage. This spectral region reflects key structural features of cellulose and hemicellulose, where vibrations at 1076 cm^−1^ and 1036 cm^−1^ correspond to the stretching of C–O–H and C–C bonds, respectively. The strong presence of these bands at 100 °C confirms that the polysaccharide backbone has not yet experienced significant cleavage [44,48]. Increasing the treatment temperature to 250 °C results in a notable attenuation of both O–H and aliphatic C–H bands, reflecting extensive dehydration, the initial breakdown of cellulose, and substantial decomposition of hemicellulose, accompanied by the release of volatile organic compounds. The marked decrease in this peak at 250 °C indicates the volatilization of carbonyl-rich side chains and the onset of condensation reactions, reflecting the selective decomposition process of hemicellulose. During the carbonization of bio-oils, carbonyl groups exhibit complex behavior, initially decreasing, reaching a minimum at a specific temperature, and subsequently increasing again [49]. Concurrently, the carbonyl peak near 1730 cm^−1^ also markedly diminishes, signifying volatilization of carbonyl-rich side chains and initiation of condensation reactions. Collectively, these changes demonstrate that early stages of oxidative thermal treatment involve the removal of primary polar functional groups and the channeling of polysaccharide segments into localized condensation pathways. At 300 °C, the spectrum undergoes a qualitative transformation. Nearly all absorption bands associated with O–H and C–H functionalities disappear, signifying almost complete elimination of hydrophilic and aliphatic components. Instead, a prominent aromatic C=C stretching vibration around 1600 cm^−1^ emerges, clearly indicating that the SCG has consolidated into a more stable, aromatic-rich structure. This aromatic band encompasses structures derived not only from lignin but also from polyaromatic domains formed through recombination of cellulose and hemicellulose decomposition products. These aromatic bands include not only structures derived from lignin but also polyaromatic structures formed through the recombination of cellulose and hemicellulose decomposition products. The stretching vibrations of aromatic C=C rings are observed in the range of 1505–1620 cm^−1^, indicating the unique aromatic characteristics associated with lignin components [50]. Taken together, these FTIR spectra delineate a clear chemical trajectory for SCG, progressing from moisture and labile functional group retention at 100 °C, through selective decomposition of hemicellulose and carbonyl group loss at 250 °C, to the complete removal of hydrophilic/aliphatic constituents and aromatic condensation at 300 °C. These transformations not only enhance the thermal stability of CSCG but also reduce polar surface functionalities, thereby lowering surface energy and suppressing continuous ice nucleation. This provides direct evidence of the anti-icing functionality of CSCG and highlights the feasibility of upcycling waste coffee grounds into sustainable fillers for advanced composite applications.

The XRD analysis results exhibit trends consistent with those observed in the FTIR spectra, revealing structural and chemical transformations at various oxidative thermal treatment temperatures. At 100 °C, only a broad amorphous peak spanning the range of 2θ = 15–25° is visible, indicating an amorphous state where cellulose, hemicellulose, and lignin are uniformly mixed without distinct crystalline features [51]. This broad peak is typically associated with reflections from the (002) planes of amorphous carbon, suggesting that a well-developed layered structure has not yet formed. Previous studies on amorphous carbon generally report a broad peak centered around 25° (2θ), reflecting the random arrangement of carbon atoms. In biomass-based carbons such as coffee grounds, it has been reported that at low temperatures below 300 °C, cellulose-like patterns with strong crystalline peaks at approximately 14°, 16°, and 23° and a weak peak near 43° are observed [52]. Extending the heating duration from 60 to 120 min at low temperatures did not induce significant changes, confirming that temperature, rather than duration, governs the structural evolution of SCG. Previous research on biomass carbonization supports this, confirming that reaction temperature predominantly governs oxidative thermal conversion processes, with reaction rates showing exponential dependence on temperature [53]. Additionally, studies aimed at improving pore volume in coffee grounds also indicated that temperature has a 3 to 7 times greater effect than increasing heating duration [54]. At 250 °C, certain peaks begin to sharpen slightly, indicating partial crystallization or structural rearrangements occurring at this intermediate stage. This is likely due to hemicellulose decomposition coupled with structural rearrangements within cellulose. At this temperature, the structure remains partially amorphous and has not yet developed into a fully crystalline phase, marking this condition as an intermediate transition. According to previous research on biochar molecular structure, heat treatment at around 250 °C prominently shows the catalytic influence of clay minerals, which can delay the decomposition of cellulose components. Hemicellulose typically begins to thermally decompose around 180 °C, reaching its maximum decomposition rate near 280 °C; thus, 250 °C falls within the primary hemicellulose decomposition region [55,56]. Upon increasing the temperature further to 300 °C, a distinct peak appears near 2θ ≈ 24°, indicative of the onset of incipient graphitic domains resulting from advanced thermal decomposition. This peak corresponds to the (002) crystallographic plane, characteristic of the early stages of incipient graphitic ordering. Previous XRD analyses of activated carbon similarly identified peaks near 23° associated with diffraction from the graphite (002) plane. The formation of a turbostratic carbon structure initially occurs with graphene-like layers arranged parallel to each other but randomly oriented between layers. During the initial stages of oxidative treatment of coffee grounds, carbon layers typically grow in this turbostratic manner [57,58,59]. Additionally, XRD patterns exhibiting two broad diffraction bands around 2θ = 23° and 43° correspond to diffraction from graphite’s (002) and (100)/(101) plane sets, respectively [53]. However, the peak typically associated with fully graphitized carbon at around 2θ ≈ 43° remains negligible, confirming that the material at 300 °C is still in an early stage of incipient graphitic ordering. This peak relates to graphite’s (100) and (101) planes and only becomes distinct in fully graphitized carbon [57]. Incipient ordering studies employing iron catalysts have shown that the appearance of graphite (002) reflections at around 750 °C indicates the onset of incipient structural ordering, achieving maximum intensity at approximately 800 °C. Thus, the temperature of 300 °C employed in this study is insufficient to achieve incipient ordering, indicating the early stages of turbostratic carbon formation. Regarding the thermal decomposition behaviors of individual biopolymer components, cellulose typically remains stable even at 300 °C, showing characteristic cellulose-like XRD patterns with strong crystalline peaks near approximately 14°, 16°, 23°, and a weaker peak around 43° [59]. These peaks diminish and become more symmetric with rising temperature. Studies on hemicellulose have reported thermal decomposition primarily occurring within the 180–340 °C range, and increasing heating rates from 10 to 50 °C/min shifts the main mass-loss temperature range slightly higher. In DTG curves, a faint shoulder peak near 205 °C suggests that hemicellulose decomposition might involve a two-step reaction mechanism [55]. These XRD results confirm that oxidative thermal treatment drives progressive structural rearrangements—transitioning from amorphous to partially ordered states, initiating turbostratic carbon formation, and reducing labile polar constituents. Such stabilization not only enhances the thermal robustness of CSCG but also underpins their anti-icing functionality by lowering surface energy and suppressing continuous ice nucleation. Overall, the results demonstrate that oxidative thermal treatment at 300 °C initiates partial ordering and reduces polar functionalities, thereby providing both enhanced stability and a structural basis for the anti-icing performance of upcycled coffee-ground fillers.

### 3.4. Pore-Structure Development Assessed by BET Analysis

**Figure 5:**
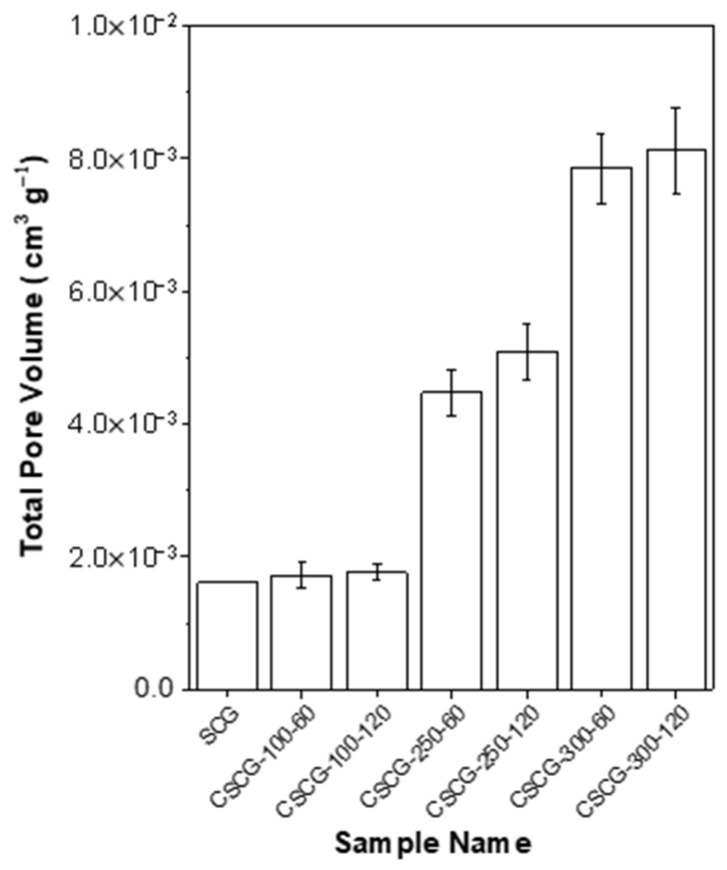
Total pore volume of SCG and CSCG prepared at different thermal treatment temperatures and durations. Sample names indicate specific thermal treatment conditions. Data was obtained from nitrogen adsorption measurements using the BET surface analysis method.

To investigate the effect of oxidative thermal treatment conditions on pore structure, total pore volumes of SCG and CSCG were measured via nitrogen adsorption using the BET method (Figure 5). The BET method is a standard technique for pore structure characterization, utilizing nitrogen adsorption at 77 K to determine specific surface area and pore volume, calculated from adsorption isotherms at relative pressures (P/P_0_) of 0.1–0.3, and total pore volume determined at P/P_0_ = 0.99 [60]. The SCG exhibited the lowest pore volume, with BET surface areas reported as low as approximately 4.4 m^2^/g, reflecting minimal structural development at low treatment temperatures like 100 °C due to insufficient thermal decomposition of major biopolymer components [61]. As the oxidative thermal treatment temperature increased, a significant increase in pore volume was observed. Notably, samples treated by oxidative thermal treatment at 300 °C exhibited markedly higher pore volumes, underscoring that temperature is a crucial factor in pore structure formation. Previous studies reported that coffee grounds subjected to oxidative thermal treatment at 300 °C for 60 min showed specific surface areas of 42.05 m^2^/g and total pore volumes of 0.14 cm^3^/g, representing increases of 141% and 76%, respectively, compared to untreated samples [62]. This sharp pore development results from the decomposition of cellulose and hemicellulose and the release of volatile compounds, promoting the transition from micropores to mesopores. Such pore evolution increases surface roughness, lowers surface energy, and thereby contributes to the anti-icing functionality of CSCG. Intermediate conditions at 250 °C showed an increase in pore volume compared to untreated SCG but were less pronounced than in samples treated at 300 °C by oxidative thermal treatment. This reflects the intermediate transition stage, consistent with the selective decomposition of hemicellulose typically occurring between 180 and 280 °C, peaking around 265 °C [63]. Longer oxidative thermal treatment durations positively influenced pore volume, yet their impact was relatively limited compared to temperature effects. This aligns with previous findings that temperature has a 3–7 times greater influence on biomass oxidative thermal conversion processes than duration. These BET results confirm that oxidative thermal treatment enhances pore structure development and supports the anti-icing functionality of CSCG. Furthermore, they highlight the feasibility of upcycling waste coffee grounds into sustainable composite fillers.

### 3.5. DOE Analysis of Mechanical and Thermal Properties

**Figure 6:**
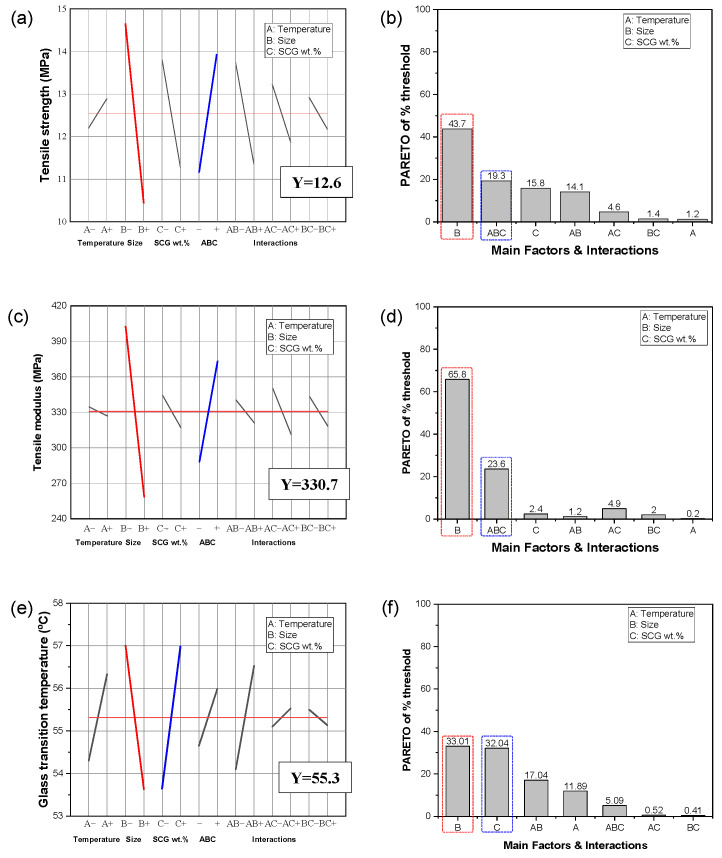
DoE and Pareto analysis of mechanical and thermal properties of polyurethane composites filled with CSCG. (**a**,**c**,**e**) Main effect plots showing the influence of oxidative thermal treatment temperature (A), particle size (B), and filler content (C) on tensile strength, tensile modulus, and glass-transition temperature, respectively. (**b**,**d**,**f**) Pareto charts of standardized effects for the same response variables, including main factors and two-factor interactions. Threshold lines indicate statistical significance.

To evaluate how the processing parameters of spent coffee grounds affect the mechanical and thermal properties of polyurethane composites, a DOE-based analysis was conducted focusing on three key factors: oxidative thermal treatment temperature (A), particle size (B), and filler content (C), with the results summarized in Figure 6. Main-effect plots (Figure 6a,c,e) illustrate the mean response at each factor level, with steeper slopes indicating stronger effects. In the tensile strength analysis (Figure 6a), particle size (B) exhibits the steepest negative slope, highlighting that smaller particles significantly enhance strength; the three-factor interaction (ABC) also notably influences strength. The relatively minor influence of oxidative thermal treatment temperature in the range of 100–300 °C on strength and modulus is attributed to the limited improvement in filler–matrix interfacial adhesion within this temperature window. Significant enhancement in strength and stiffness generally requires structural evolution beyond low-temperature oxidative thermal treatment, such as incipient structural ordering (turbostratic carbon formation), which was not observed at 300 °C. Therefore, the internal particle characteristics are considered to have changed only slightly with temperature. Achieving complete graphitic ordering typically requires substantially higher oxidative thermal treatment temperatures. For tensile modulus (Figure 6c), both particle size (B) and filler content (C) are important, though the magnitude of B is smaller than in strength. T_g_ (Figure 6e) is again predominantly influenced by particle size and filler content, with minor effects from the AB interaction. This is likely because physical properties such as filler size and content directly affect the polymer matrix’s molecular mobility and free volume. The Pareto charts (Figure 6b,d,f) quantify standardized effects and illustrate statistical significance through reference lines. Particle size (B) is the most significant factor affecting tensile strength and modulus, along with notable ABC interactions. For T_g_, particle size remains dominant, closely followed by filler content (C). The comparatively modest impact of oxidative thermal treatment temperature within the 100–300 °C range on strength and modulus is attributed to limited improvements in filler-matrix interfacial adhesion. Structural enhancements such as incipient structural ordering typically require higher temperatures; thus, internal particle characteristics remained largely unchanged within this temperature range. These DOE results confirm that particle size and filler content are the primary factors dictating composite properties, while temperature effects within 100–300 °C remain modest. Nonetheless, oxidative thermal treatment at higher levels promotes morphological changes that enhance anti-icing functionality. These findings highlight the potential of upcycling waste coffee grounds into sustainable fillers for polyurethane composites in advanced surface-protection applications.

### 3.6. Surface Wettability and Anti-Icing Performance of PU/SCG-Based Composites

**Figure 7:**
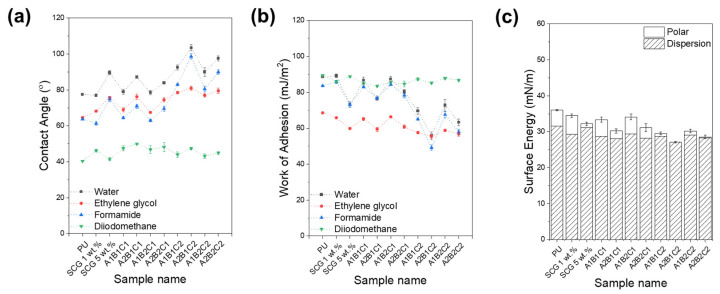
Effect of filler formulation on surface wettability and interfacial properties of PU composites incorporating CSCG. (**a**) Water contact angle and (**b**) work of adhesion of PU/CSCG composites as a function of different formulations. (**c**) Total surface energy and polar component of surface energy.

This study comprehensively evaluated the surface characteristics and anti-icing performance of PU composites incorporating SCG and CSCG as fillers (Figure 7). Pure PU displayed moderate hydrophobicity (77.4°), consistent with previous studies. Low concentrations of untreated SCG (1 wt.%) minimally altered the contact angle (77.0°), while higher concentrations (5 wt.%) significantly increased hydrophobicity (89.6°). This is attributed to the organic compounds such as fatty acids, lignin, cellulose, and hemicellulose present in coffee grounds, promoting surface hydrophobization. Composites containing CSCG exhibited significantly increased hydrophobicity, particularly at higher oxidative thermal treatment temperatures (A_2_, 300 °C) due to the substantial reduction in polar functionalities, reducing surface energy. The A_2_B_1_C_2_ sample demonstrated the highest water contact angle (103.5°), negligible polar components (0.01 mN/m), and markedly reduced total surface energy from 36.00 m·N/m (pure PU) to 27.13 mN/m, evidencing significant surface chemical modifications enhancing hydrophobicity. Smaller particle sizes (B_1_, 100 µm) and higher filler content (C_2_, 5 wt.%) enhanced surface roughness, effectively inducing a Cassie–Baxter hydrophobic state. Surface roughness is one of the most effective factors influencing surface properties such as hydrophobicity, and its effect on the hydrophobicity of periodically roughened PE and PVC surfaces has been demonstrated through molecular dynamics simulations [64,65]. While hydrophobicity increased on all rough surfaces, the structured PE surface exhibited a greater increase in contact angle compared to the corresponding PVC surface, as the flat PE surface already had a higher intrinsic contact angle. Work of adhesion analysis showed the lowest adhesion value for the A_2_B_1_C_2_ composite (55.81 mJ/m^2^) compared to pure PU (88.68 mJ/m^2^), clearly correlating enhanced hydrophobicity with improved anti-icing performance. The combination of reduced polar functionalities, increased surface roughness, and lowered surface energy demonstrates that CSCG incorporation promotes ice-nucleation suppression and discontinuous ice coverage, underscoring both the anti-icing performance and the sustainability potential of upcycling waste coffee grounds as functional fillers.

### 3.7. Microstructural Characteristics of PU/CSC CFRP Composites

**Figure 8:**
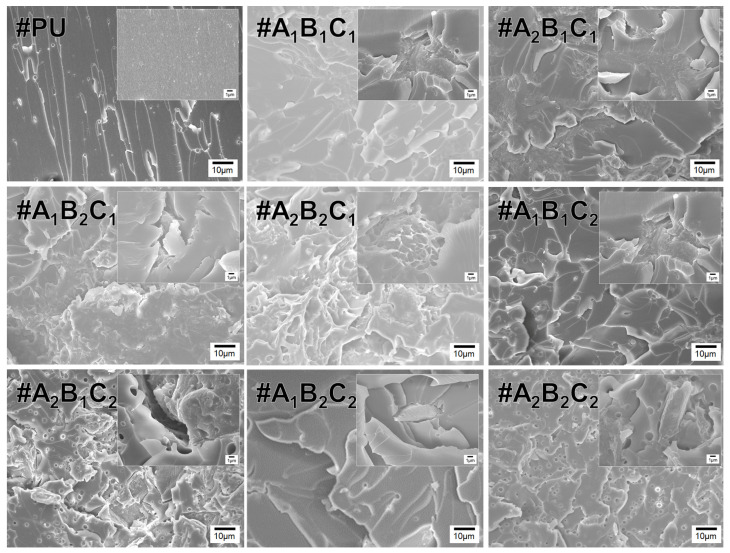
SEM images of CFRP composites coated with PU incorporating CSCG. Images highlight surface morphology and interfacial adhesion between the PU coating and CFRP substrate. Samples were prepared with different CSCG formulations to evaluate filler dispersion and coating uniformity.

Figure 8 presents SEM micrographs of CFRP panels coated with PU containing CSCG. These images clearly reveal the evolution of porosity and microstructure as a function of oxidative thermal treatment temperature (A), particle size (B), and filler content (C). CFRP composites consist of carbon fibers, a polymer matrix, and the interfaces between these components, with the microstructure, chemical composition, bonding characteristics, and interfacial adhesion strength greatly influencing their mechanical properties and fracture behavior. Even neat PU exhibits minor matrix porosity, primarily due to resin shrinkage and volatile release during the curing process. Such porosity defects formed during manufacturing adversely affect both the static and dynamic mechanical properties of the composites [66]. At low filler loading (1 wt.%; A_1_B_1_C_1_, A_2_B_1_C_1_, A_1_B_2_C_1_, A_2_B_2_C_1_), CSCG particles were uniformly dispersed, enabling adequate resin infiltration and resulting in minimal pore formation. Specifically, lower carbonization temperature (100 °C, A_1_) and smaller particle size (100 µm, B_1_) effectively suppressed particle agglomeration and resin starvation, maintaining excellent interfacial properties. Conversely, high filler loading (5 wt.%; #A_1_B_1_C_2_, #A_2_B_1_C_2_, #A_1_B_2_C_2_, #A_2_B_2_C_2_) significantly reduced inter-particle spacing, promoting agglomeration and leading to extensive porosity. This issue was particularly severe under the high-temperature and large-particle-size combination (#A_2_B_2_C_2_, 300 °C, 300 µm). Under these conditions, resin excessively penetrated the highly porous fillers but became insufficient on the outer surfaces, creating resin-starved areas and leading to poor impregnation, reduced stress-transfer efficiency, and lower mechanical performance. In polymer composites, the inclusion of fillers tends to induce redistribution of concentration as particle size increases, leading to filler-depleted regions near the wall [67]. This phenomenon has been observed more prominently in highly filled and semi-crystalline polymers compared to amorphous ones [68]. In a study by Samal, it was reported that aggregation and sedimentation typically initiate with larger filler particles having a specific surface area of 5–12 m^2^/g, although the extent of these effects depends on the particle size distribution [69]. In vacuum infusion processes, such resin-starved regions are considered critical manufacturing defects known to negatively impact fatigue and static properties. However, resin penetration into CSCG micropores can positively enhance interfacial adhesion and thermal conductivity. Hence, optimizing the carbonization temperature, particle size, and filler content is crucial to balancing mechanical integrity, thermal performance, and anti-icing efficacy. These SEM results emphasize that controlling processing parameters not only improves structural robustness but also enhances the anti-icing functionality of CSCG-based coatings, highlighting the potential of upcycling waste coffee grounds into sustainable composite fillers.

### 3.8. Mechanical Performance and Anti-Icing Correlation of PU/CSCG Composites

**Figure 9:**
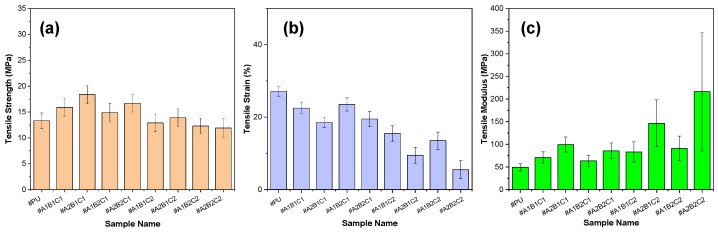
Mechanical properties of PU composites incorporating SCG and CSCG: (**a**) tensile strength, (**b**) tensile strain, and (**c**) tensile modulus as functions of filler content and thermal treatment conditions.

In this study, PU composites reinforced with CSCG were systematically evaluated to elucidate the correlation between mechanical performance and anti-icing properties, based on varying oxidative thermal treatment conditions, including oxidative thermal treatment temperature, particle size, and filler loading (Figure 9). At lower oxidative thermal treatment temperatures (100 °C, A_1_), CSCG particles underwent limited oxidative thermal treatment, producing relatively softer and less porous structures. This minimally affected the flexibility of the PU matrix, resulting in slight improvements in tensile strength and modulus compared to neat PU. At temperatures below 300 °C, cellulose remains thermally stable, indicating that cellulose-like structures are preserved under these conditions [70,71]. In contrast, higher oxidative thermal treatment temperatures (300 °C, A_2_) produced CSCG particles that were structurally rigid and significantly more porous. This led to a substantial increase in interfacial contact area and bonding strength with the PU matrix, resulting in marked improvements in tensile strength and modulus, albeit with a notable reduction in flexibility. Among studies on coffee ground oxidative thermal treatment, some have reported that samples treated at 300 °C exhibited higher specific surface areas than those thermally treated at 600 °C under argon, which was associated with an increase in surface carboxyl groups. However, low-temperature treatment at 100 °C has been shown to have only a minimal effect on the development of pore structures in coffee grounds [72]. Particle size also exerted a significant impact on composite mechanical properties. Smaller particle size (100 µm, B_1_) promoted uniform dispersion of CSCG within the PU matrix, thereby increasing the interfacial contact area and leading to robust composite structures with improved tensile strength and modulus, accompanied by substantial strain reduction. Conversely, larger particle size (300 µm, B_2_) increased inter-particle spacing and particle agglomeration, which limited uniform dispersion, thus restricting improvements in tensile strength; however, these larger spaces facilitated strain absorption, resulting in less severe strain reduction compared to smaller particle size conditions. Filler loading also proved critical, where lower filler contents (1 wt.%, C_1_) resulted in excellent dispersion within the PU matrix, significantly improving tensile strength and modulus while minimizing reductions in flexibility. However, higher filler loading (5 wt.%, C_2_) led to notable particle agglomeration and increased porosity, limiting tensile strength gains and drastically reducing strain. This effect was more pronounced at higher oxidative thermal treatment temperatures. Considering these combined effects, the optimized composite formulation (A_2_B_1_C_2_) comprising high oxidative thermal treatment temperature (300 °C), small particle size (100 µm), and high filler content (5 wt.%) provided the maximum structural rigidity, demonstrating significant potential for applications requiring superior resistance to environmental stresses and effective ice prevention. Highly hydrophobic and icephobic-like PU coatings have been reported to offer excellent mechanical strength, durability, adhesion, and good abrasion resistance. However, a limitation of PU-based coatings lies in their inherently hydrophilic nature, as they contain numerous polar groups on the surface [73]. Conversely, applications demanding enhanced flexibility may benefit from lower filler content and smaller particle size combinations (A_1_B_1_C_1_, A_1_B_2_C_1_). The observed correlations between mechanical properties and anti-icing performance are closely linked to surface properties and microstructural characteristics. Enhanced hydrophobicity and surface roughness synergistically contributed to improved anti-icing functionality. Consequently, PU/CSCG composites represent highly promising anti-icing coating materials for aircraft, wind turbines, and infrastructure exposed to extreme environmental conditions. In addition to the tensile data, the observed reduction in strain at break suggests increased brittleness, which is typical of filled polymer systems. This behavior can be attributed to stress concentration, limited particle dispersion, and weak filler–matrix adhesion, which collectively reduce the deformability of the composite. Similar embrittlement mechanisms in particle-reinforced polymers have been widely reported in the literature, where particle size, filler distribution, and interfacial bonding were identified as key factors controlling fracture behavior [74,75]. Although fracture-surface SEM and adhesion-strength measurements were not performed in this study, our results are consistent with these prior observations, supporting the proposed interpretation of filler-induced brittleness.

### 3.9. Thermal Stability and Glass-Transition Behavior of PU/CSCG Composites

**Figure 10:**
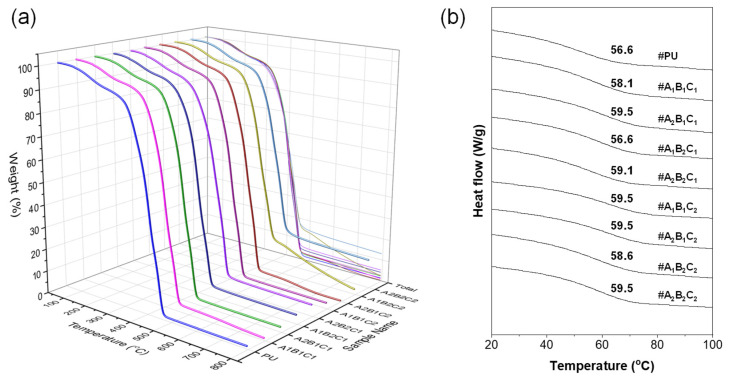
Thermal properties of polyurethane composites filled with CSCG: (**a**) TGA curves showing thermal stability and residual weight; (**b**) DSC curves showing variation in glass-transition temperature with filler incorporation (Composite samples were tested under N_2_ atmosphere from room temperature to 600 °C).

The thermal stability and glass-transition behaviors of PU composites incorporating CSCG were comprehensively evaluated using TGA and DSC (Figure 10). TGA curves clearly demonstrated that neat PU exhibited rapid mass loss beginning around 250 °C, completing major degradation near 400 °C, primarily due to extensive thermal decomposition of PU polymer chains converting into volatile species. Typically, thermal decomposition of polyurethane initiates with urethane bond cleavage in the temperature range of 150–250 °C. Aromatic diisocyanate-based TPUs generally show higher thermal stability compared to aliphatic counterparts. In contrast, PU composites containing CSCG exhibited a significantly delayed onset of mass loss, indicating enhanced thermal stability relative to neat PU. The incorporation of CSCG modifies internal heat transfer pathways or elevates the initial decomposition temperature, thus effectively delaying early thermal degradation. Particularly, CSCG particles produced at higher carbonization temperatures (300 °C, #A_2_) have high carbon content and well-developed microporous structures, which strongly interact with the PU matrix, effectively forming a thermal-barrier network that retards thermal decomposition even at elevated temperatures. Increasing the filler loading from 1 to 5 wt.% further enhanced thermal resistance by increasing the fraction of thermally stable CSCG particles, consequently increasing the residual mass fraction at high temperatures. Although smaller particle size (100 µm, #B_1_) provided increased surface area to facilitate improved dispersion and interfacial interactions within the PU matrix, the impact of particle size on thermal stability was relatively moderate compared to carbonization temperature or filler content. The DSC analysis distinctly showed a clear thermal transition (T_g_) at relatively lower temperatures for neat PU, driven by rapid mobility increases of polymer chains transitioning from the glassy to the rubbery state. Composites containing CSCG demonstrated a gradual increase in T_g_ compared to neat PU due to CSCG particles restricting molecular mobility within the PU matrix through physical and chemical interactions. Particularly, the composite formulated at high carbonization temperature (300 °C, #A_2_), small particle size (100 µm, #B_1_), and high filler content (5 wt.%, #C_2_) exhibited the most significant shift in T_g_ to higher temperatures. This pronounced effect arises because the highly porous structure of CSCG forms strong interfacial adhesion with PU, effectively constraining polymer chain mobility. Furthermore, smaller particle sizes significantly increase interfacial contact area, while higher filler loading further amplifies chain mobility restrictions, cumulatively enhancing the T_g_ shift. Additionally, penetration of PU resin into CSCG micropores provides mechanical interlocking and chemical interactions, substantially improving interfacial adhesion.

### 3.10. Anti-Icing Performance of PU/SCG- and PU/CSCG-Coated CFRP Composites

**Figure 11:**
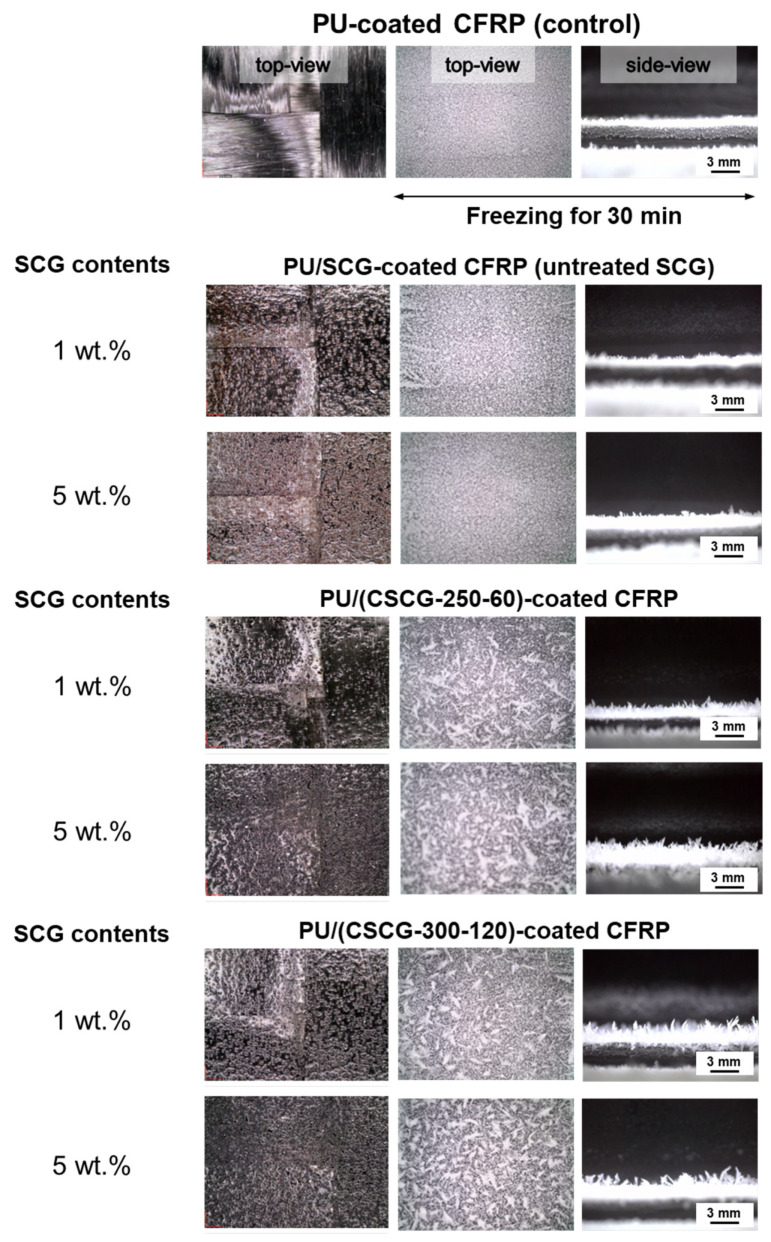
Anti-icing behavior of PU-coated CFRP composites containing SCG and CSCG at different filler contents and thermal treatment conditions. Each sample was subjected to freezing at −30 °C for 30 min prior to evaluation. Top-view and side-view images compare anti-icing performance across filler types (untreated SCG, CSCG-250-60, CSCG-300-120) and contents (1 and 5 wt.%).

To assess anti-icing performance, PU composites containing untreated SCG or CSCG at 1 wt.% and 5 wt.% were applied to CFRP panels, and their behaviors upon freezing were examined (Figure 11). After freezing for 30 min at 0 °C, the neat PU reference clearly showed a uniformly dense ice layer in both top- and side-view images, indicating the poorest anti-icing performance. This is primarily attributed to the inherently hydrophilic nature of PU coatings, arising from high surface energy and abundant polar functional groups, which strongly promote ice adhesion. Incorporation of untreated SCG led to an increase in ice layer thickness, particularly at the 5 wt.% loading, producing a thicker, continuous sheet of ice, confirming only marginal improvement in anti-icing capability. This limited improvement results from polar components, such as cellulose and hemicellulose, present in untreated SCG, facilitating strong hydrogen bonding and enhancing ice nucleation and adhesion. In contrast, PU composites containing CSCG demonstrated distinct improvements dependent on oxidative thermal treatment conditions. At 1 wt.% filler loading, CSCG-250-60 effectively suppressed ice nucleation, producing sparse and discontinuous crystal patterns. More significantly, CSCG-300-120 further reduced crystal size and density, resulting in the most effective anti-icing performance. These effects can be attributed to significant reductions in polar functionalities and enhanced micro-scale roughness formed during the oxidative thermal treatment process, inducing a stable Cassie–Baxter hydrophobic state. Increasing filler content to 5 wt.% slightly increased apparent ice thickness in side-view observations; however, both CSCG formulations still fragmented the ice layer into irregular and loosely interconnected domains. Although ice thickness appeared to increase slightly in side-view observations, this effect was primarily associated with the treatment temperature rather than filler loading. Specifically, samples treated at 250 °C showed relatively denser and more continuous ice coverage compared to those treated at 300 °C, where ice formation was significantly suppressed. At higher treatment temperatures, the markedly reduced surface energy limited interfacial heat transfer, keeping the coating surface relatively warmer and thereby suppressing continuous ice nucleation and growth. In contrast, neat PU and composites prepared with untreated SCG retained higher surface energy, which facilitated faster heat transfer, accelerated surface cooling, and consequently promoted more extensive icing. While such differences were not always obvious from side-view observations alone, they became clear in the top-view images, where CSCG-300-120 coatings displayed fragmented and discontinuous ice coverage in contrast to the relatively continuous ice layers observed for CSCG-250-60 and neat PU. Particularly, the CSCG-300-120 sample exhibited the most pronounced effect, with its oxidatively treated, micro-roughened surface effectively hindering ice adhesion and lateral growth. These results clearly indicate that optimizing oxidative thermal treatment temperature and dwell time greatly enhances the surface chemistry and roughness of CSCG, thereby contributing to improved anti-icing functionality of PU coatings. Despite localized increases in ice layer thickness at higher filler loadings, the overall ice adhesion and continuity were notably reduced. Consequently, PU/CSCG composites show promising potential for use as anti-icing coatings in aircraft, wind turbines, and infrastructure operating under extreme environmental conditions, while also suggesting economic and environmental benefits through the upcycling of waste coffee grounds into sustainable functional fillers.

## 4. Conclusions

This study comprehensively evaluated mechanical, thermal, and anti-icing properties of PU composites reinforced with carbonized CSCG. CSCG oxidatively treated at 300 °C for 120 min (A_2_) significantly enhanced hydrophobicity (103.5° water contact angle) and notably reduced adhesion work (55.81 mJ/m^2^) by nearly eliminating polar surface functionalities. XRD and FTIR analyses confirmed the development of incipient turbostratic ordering and microporous structures (BET pore volume: 0.14 cm^3^/g), creating an effective thermal barrier network that improved thermal stability and significantly raised the T_g_. DOE analysis revealed particle size (B) as the most critical parameter influencing tensile strength, modulus, and T_g_, with smaller particles (100 µm) achieving optimal mechanical performance. The optimized composite formulation (A_2_B_1_C_2_) simultaneously maximized structural rigidity and anti-icing efficiency, showing promising potential for applications in aircraft, wind turbines, and infrastructure operating under extreme environmental conditions. Furthermore, this approach exemplifies a sustainable, environmentally friendly method to convert biomass waste into high-value functional materials.

## Data Availability

The original contributions presented in this study are included in the article. Further inquiries can be directed to the corresponding authors.
